# New Treatment Options for Hyperkalemia in Patients with Chronic Kidney Disease

**DOI:** 10.3390/jcm9082337

**Published:** 2020-07-22

**Authors:** Pasquale Esposito, Novella Evelina Conti, Valeria Falqui, Leda Cipriani, Daniela Picciotto, Francesca Costigliolo, Giacomo Garibotto, Michela Saio, Francesca Viazzi

**Affiliations:** Clinica Nefrologica, Dialisi, Trapianto, Department of Internal Medicine, University of Genoa and IRCCS Ospedale Policlinico San Martino, Viale Benedetto XV, 16132 Genoa, Italy; novella.conti@hsanmartino.it (N.E.C.); valeria.falqui@hsanmartino.it (V.F.); cipriani.leda@gmail.com (L.C.); danipicciotto@me.com (D.P.); 3232594@studenti.unige.it (F.C.); gari@unige.it (G.G.); michela.saio@virgilio.it (M.S.); francesca.viazzi@unige.it (F.V.)

**Keywords:** hyperkalemia, chronic kidney disease, heart failure, sodium polystyrene sulfonate, patiromer, sodium zirconium cyclosilicate

## Abstract

Hyperkalemia may cause life-threatening cardiac and neuromuscular alterations, and it is associated with high mortality rates. Its treatment includes a multifaceted approach, guided by potassium levels and clinical presentation. In general, treatment of hyperkalemia may be directed towards stabilizing cell membrane potential, promoting transcellular potassium shift and lowering total K^+^ body content. The latter can be obtained by dialysis, or by increasing potassium elimination by urine or the gastrointestinal tract. Until recently, the only therapeutic option for increasing fecal K^+^ excretion was represented by the cation-exchanging resin sodium polystyrene sulfonate. However, despite its common use, the efficacy of this drug has been poorly studied in controlled studies, and concerns about its safety have been reported. Interestingly, new drugs, namely patiromer and sodium zirconium cyclosilicate, have been developed to treat hyperkalemia by increasing gastrointestinal potassium elimination. These medications have proved their efficacy and safety in large clinical trials, involving subjects at high risk of hyperkalemia, such as patients with heart failure and chronic kidney disease. In this review, we discuss the mechanisms of action and the updated data of patiromer and sodium zirconium cyclosilicate, considering that the availability of these new treatment options offers the possibility of improving the management of both acute and chronic hyperkalemia.

## 1. Introduction

Potassium (K^+^) is a key element in body physiology. It regulates many biological processes, such as acid–base homeostasis, hormone secretion, systemic blood pressure control and gastrointestinal motility [1]. However, probably the most important role of K^+^ is its participation in generating bioelectricity, by establishing ion gradients and flows between the extracellular and intracellular spaces, thus regulating resting membrane potential and cellular excitability, which are essential to the function of excitable tissues, such as nerve, muscle and cardiac conduction tissues.

This function is a consequence of the high compartmentalization of K^+^, due to the ubiquitous presence of plasma membrane Na-K-ATPases, which pump sodium out of, and K^+^ into, the cell [2]. Therefore, K^+^ results from the most concentrated intracellular electrolyte, while its extracellular concentration is extremely low. We estimate a total K^+^ body content of approximately 50 mEq/ kg (i.e., 3500 mEq in a 70-kg person); about 98% of this K^+^ is within cells, while only 2% (70 mEq) is in the extracellular fluid, where it reaches normal concentrations of 3.5 to 5.0 mmol/L [3].

Hyperkalemia is defined as a serum potassium level greater than 5.0 mmol/L, while severe hyperkalemia is defined as a level greater than 6.0 mmol/L. It is a very common disorder, and in the United States more than 800,000 emergency department (ED) visits occur annually because of hyperkalemia [4]. The actual incidence and prevalence of hyperkalemia in the general population are unknown, but studies based on large cohorts have reported incidence rates between 1 and 3 per 100 persons per year, rising to 10% in hospitalized patients [5,6].

Moreover, hyperkalemia prevalence may be significantly high in the presence of certain predisposing conditions. So, although the available data are not uniform [7,8], an analysis of a large geographically diverse population showed basal potassium values of ≥ 5.0 mmol/L in 9.1% of patients with chronic heart failure (CHF), in 11.5% of chronic kidney disease (CKD) stage 3–5 patients, in 8.3% of patients with diabetes, and in 13.1% of those patients with all these conditions [9]. In addition, among CKD patients, those requiring dialysis represent a group at particularly high risk of hyperkalemia [10].

Clinical complications and death in hyperkalemia patients are mainly determined by the cardiac electrophysiological effects of elevated K^+^ levels [11]. Indeed, hyperkalemia, by diminishing the K^+^ intracellular/K^+^ extracellular ratio, reduces the membrane potential, causing a partial depolarization of the cell membrane, which results in an initial increase in conduction velocity. Then, if persistent and profound, hyperkalemia also decreases membrane excitability by the inactivation of the voltage-gated sodium channels, making the cell refractory to excitation, and thus leading to arrhythmias and heart block [12]. Moreover, besides cardiac effects, hyperkalemia can also cause other physiologic perturbations, such as muscle weakness progressing to flaccid paralysis, and metabolic acidosis, which in turn may contribute to the progression of CKD [13].

The treatment of hyperkalemia may involve the recognition different time-points and goals, guided by potassium levels and the severity of the clinical presentation. In general, the first aim is to prevent cardiac consequences and lower serum potassium to safe levels as soon as possible; then it is important to reduce the K^+^ body content, aiming to maintain serum potassium at normal values [14]. The latter can be obtained by dialysis, or by increasing potassium elimination via urine or the gastrointestinal tract. For a long time, the only therapeutic option for increasing fecal K^+^ excretion has been represented by sodium polystyrene sulfonate, a cation-exchanging resin the efficacy and safety of which have been questioned. Recently, new drugs able to promote gastrointestinal potassium elimination, namely patiromer and sodium zirconium cyclosilicate, have been developed and studied in large trials, proving their efficacy and safety in different clinical contexts. In this review, we briefly discuss the pathophysiology of potassium homeostasis and hyperkalemia, focusing attention on the mechanisms of action and the clinical data of patiromer and sodium zirconium cyclosilicate, considering that these new treatments may represent a chance to improve the management of both acute and chronic hyperkalemia.

## 2. Potassium Homeostasis: An Overview

Due to its important functions, and considering that large deviations in K^+^ serum levels are not compatible with life, K^+^ homeostasis is finely regulated by numerous mechanisms.

Classically, we distinguish between external and internal K^+^ balance [3]. External K^+^ balance regulates K^+^ body content, and it is the result of the relationship between K^+^ assumption (by diet or other sources, such as infusions) and K^+^ excretion, which is a function of the kidney and the gut. Internal balance accounts for K^+^ distribution across cell compartments, which can be influenced by several factors and may be important in determining the actual K^+^ extracellular level. 

The external potassium balance is mainly influenced by K^+^ excretion via the kidney. The normal kidney has a large capacity to excrete potassium and maintain a normal serum potassium concentration. Potassium is freely filtrated by the glomerulus, and is then reabsorbed by the proximal tubule and thick ascending limb, such that only a small amount reaches the aldosterone-sensitive distal nephron, where K^+^ excretion, coupled with sodium reabsorption, is finely regulated [15].

The main factors modulating renal K^+^ excretion are sodium delivery to the distal nephron, K^+^ serum levels and aldosterone plasma concentration. In particular, a relevant role in regulating K^+^ homeostasis is played by the adrenal glands, where aldosterone is synthesized, in a negative feedback loop in response to high K^+^ levels [16].

Interestingly, evidence is emerging concerning the role of the central nervous system in influencing circadian variability in relation to potassium excretion [1]. Apart from the kidneys, the gastrointestinal tract also contributes to K^+^ excretion. In healthy subjects, this contribution is minimal (about 10% of the total), while in the case of renal disease it may increase until it accounts for 50% of the total potassium excretion in patients on dialysis [17]. However, these systems are strictly related, and recently it has been shown that the K^+^ enteral load may influence renal excretion, suggesting the presence of a gut-dependent kaluresis, the mechanisms of which are still under investigation [18]. 

Complementarily to the external K^+^ balance, the internal mechanisms of K^+^ distribution are very important in regulating K^+^ homeostasis and extracellular levels. The physiological factors involved in modulating the shifting of potassium into the cells include the acid–base balance, insulin, and beta-adrenergic stimulation [19]. In particular, metabolic acidosis induces K^+^ shift from the intra-to the extracellular space, while the opposite is mediated by insulin and beta-adrenergic signaling. However, external and internal K^+^ regulatory mechanisms are integrated, and need to always be active in order to maintain K^+^ homeostasis.

A western diet typically contains approximately 50–100 mEq (2–4 g), while a potassium intake of about 90–120 mEq/day (3.5–4.5 g) is recommended [20]. Consequently, the potassium content in a meal may be higher than the potassium present in the plasma, so compensatory mechanisms are necessary in order to avoid a rapid rise in extracellular K^+^ levels. For example, after a potassium-rich meal, the K^+^ transcellular shift into the cells is suddenly activated, until the kidney reestablishes total body potassium content trough the adjustment of renal potassium excretion.

Knowledge of the basal physiologic regulators of external and internal K^+^ homeostasis is necessary in order to understand the main clinical conditions leading to K^+^ dysregulation and the appropriate therapeutic approaches, providing, at the same time, a rational basis for developing new drugs [21].

## 3. Hyperkalemia: Physiopathology, Risk Factors, Clinical Consequences

Hyperkalemia may be caused by several conditions that may alter K^+^ homeostasis [1]. First, it could be the consequence of an increased K^+^ body content due to excessive K^+^ intake, or, more commonly, due to reduced renal excretion. Renal K^+^ excretion may be impaired as a result of advanced renal damage. Indeed, while the normal kidney presents adaptation mechanisms that preserve potassium homeostasis, the diseased kidney has a much lower capacity for handling acute potassium loads [22]. Failures of the kidneys in regulating the potassium balance may result from multiple factors, including a reduced glomerular filtration rate, decreased distal delivery of sodium, intrinsic abnormalities of the distal nephron, and decreased mineralocorticoid activity (e.g., hypoaldosteronism), which impair the capacity of the distal nephron to eliminate K^+^ from the urine. Moreover, concomitant metabolic alterations, such as acidemia and hyperglycemia, may also concur [23].

Hypoaldosteronism, in turn, may be caused by diabetes, adrenal disease, numerous drugs (e.g., nonsteroidal anti-inflammatory drugs, beta-blockers, inhibitors of the renin-angiotensin-aldosterone system-RAASi, mineralocorticoid receptor blockers, calcineurin-inhibitors, etc.) and old age [24]. Beyond an increase of K^+^ body content, alterations in the K^+^ distribution across cell compartments can also lead to hyperkalemia. These conditions determine the net release of potassium from damaged cells, such as in cases of trauma, rhabdomyolysis or hemolysis. Moreover, an impaired distribution of K^+^ between the intracellular and extracellular spaces can also be due to metabolic acidosis, decompensated diabetes or dysfunctions of the autonomic nervous system.

Considering the physiopathology of potassium homeostasis, it is not surprising that advanced age, chronic kidney disease (CKD), chronic heart failure (CHF), diabetes, use of RAASi (such as ACE-inhibitors-ACEi and angiotensin receptor blockers-ARB) and mineralocorticoid receptor blockers (MRA) constitute the main risk factors in the development of hyperkalemia [9].

However, a special consideration must be given to the risk of hyperkalemia linked to the use of RAASi and MRA. Indeed, these drugs, because of the evidence of their morbidity and mortality benefits, are widely prescribed to fragile patients, such as patients with diabetes, CKD and CHF [25]. Several studies have evaluated the risk of hyperkalemia associated with RAASi therapy. For example, in the Stockholm Creatinine Measurements (SCREAM) project, 69,426 new users of ACEi/ARB therapy were followed for one year. Overall, hyperkalemia occurred in 1.7% of the entire cohort, but its incidence rose to 29% in patients with severe CKD [26]. Moreover, the risk of hyperkalemia seems further increased when combined RAASi therapy is prescribed. In the Veterans Affairs Nephropathy in Diabetes (VA NEPHRON-D) study, designed to assess the safety of RAASi for type II diabetic kidney disease patients, hyperkalemia was observed in 18.4% of the patients on losartan monotherapy, and in 31.5% of those on a combination therapy with lisinopril [27].

Following the results of these and other studies, the combination of ACEi and ARB is no longer recommended [28]. Similar results have been observed among patients treated with the combination of RAASi and MRA, which, although effective in improving clinical outcomes, may expose patients to a high risk of hyperkalemia [25,29].

The early recognition and treatment of hyperkalemia is essential, because this condition, although often clinically silent, may have severe consequences. Hyperkalemia is associated with poor outcomes and high mortality rates, both in the general population and in different clinical settings, including patients with cardiac and renal diseases and critically ill patients [30,31]. Moreover, hyperkalemia has been described as an independent predictor of mortality in patients admitted to the ED [32].

## 4. Hyperkalemia: Treatment Strategies

From the pathophysiological point of view, the therapeutic approaches to hyperkalemia can have three different targets: (i) cell membrane potential stabilization; (ii) shifting potassium from extracellular spaces into the cells (i.e., acting on internal K^+^ balance); and (iii) lowering K^+^ levels and enhancing potassium elimination (i.e., acting on external K^+^ balance).

Membrane stabilization may be achieved through the administration of intravenous calcium (calcium chloride or calcium gluconate), while potassium redistribution may be promoted using insulin/glucose, beta-adrenergic agonists (such as albuterol and salbutamol, both intravenous and inhaled) and sodium bicarbonate [33]. These treatments are often preferred in emergency interventions, since they can reduce K^+^ levels within a few minutes [34]. However, while they act rapidly, their effects also fade very rapidly.

So, complementary to the strategies that promote the shifting of potassium into cells, the reestablishment of potassium homeostasis should include the reduction of body K^+^ content.

This can be achieved through the limitation of potassium intake and the use of medications that increase potassium elimination via urine or the gastrointestinal tract (GI), such as loop diuretics or cation-exchanging resins, or alternatively, by use of hemodialysis, which can reduce body K^+^ content but usually require more time to act [35]. 

In particular, the use of drugs increasing GI potassium elimination is valuable in patients with advanced CKD, who, as discussed above, present significant fecal K^+^ excretion.

Although these treatments are widely used in clinical practice, it should be recognized that, as also shown by a recent Cochrane review, standardized therapeutic protocols do not exist [36]. So, in a prospective multicenter study exploring real-life hyperkalemia management in many EDs, it has been shown that there was a great heterogeneity among the different sites, while, even if insulin/glucose was the most common therapy employed, in the majority of the patients multiple treatments were prescribed [37].

## 5. The “Old-Fashioned” Sodium Polystyrene Sulfonate

Among the different possibilities for treating hyperkalemia, recently, attention has been focused on the GI elimination of potassium, mainly because of the availability of new drugs, such as patiromer and sodium zirconium cyclosilicate.

Indeed, historically, the only options for promoting K^+^ elimination by the GI have been limited to the “old” cation-exchanging resin, sodium polystyrene sulfonate (SPS), and its derivate calcium polystyrene sulfonate. SPS is a benzene, diethenyl-polymer with ethenylbenzene-sulfonated sodium salt, whose reactive sulfonic groups exchange preloaded sodium for K^+^ along the GI lumen (mostly in the large intestine) [38]. It can be given orally or as an enema, and it is often given with sorbitol to prevent constipation. Theoretically, the exchanging capacity of SPS is 1 mmol of potassium per 1 g of resin, but its efficiency in vivo may be lower than expected because sodium release is only partial [39]. Moreover, the peak effect is seen 4–6 h after the administration, and this is the reason why SPS is not indicated as an emergency intervention for hyperkaliemia [40].

SPS was approved for treatment of acute hyperkalemia in 1958, but surprisingly, despite its common use, its safety and efficacy have been poorly studied in controlled studies [41].

In 2011, analyzing a recent retrospective cohort of 122 patients (38% with CKD), Kessler et al. documented a possible direct dose–response relationship between SPS (used at a dose of 15, 30, 45 and 60 g) and a reduction in serum potassium [42]. Similarly, in 2015, in a randomized placebo-controlled trial involving 33 CKD patients with mild hyperkalemia, Lepage et al. found that SPS was superior to the placebo in reducing serum potassium over 7 days in patients with mild hyperkalemia and CKD [43]. So, these data confirm the clinical practice of using SPS as a part of the treatment of hyperkalemia.

The adverse effects of SPS include electrolyte disturbances, such as hypokalemia and hypomagnesemia, and gastrointestinal symptoms, such as nausea, constipation and diarrhea. However, severely adverse gastrointestinal effects, including ulceration, bleeding, ischemic colitis and perforation have also been reported, especially when combined with sorbitol [44]. In a retrospective case-control study including 123,391 inpatients, of whom 2194 were prescribed SPS, there was a doubling of the incidence of colonic necrosis between SPS users and non-users, which was not significant (0.14% vs. 0.07%, *P* = 0.2) [45]. Interestingly, a large retrospective population-based study from Canada similarly documented a significant two-fold increase in the incidences of hospitalization for serious adverse GI events in 20,020 SPS users, when compared with matched non-users [46]. Moreover, Laureati et al. examined SPS use and GI safety in a cohort including 3690 adults with CKD stages 4–5 (1288 on chronic dialysis) naive to SPS. They found that SPS initiation was associated with a higher incidence of severe GI adverse events, mainly ulcers and perforations, in a probable dose-dependent manner [47]. Beyond the GI side effects, it should be underlined that significative drug interactions have also been described while using SPS, and this could be relevant for cardiac and renal patients, who often take multiple pharmacological therapies [48].

Evaluating all these potential detriments associated with the chronic use of SPS, currently, the Food and Drug Administration (FDA) recommends the avoidance of SPS prescription for patients with active GI diseases or with a history of recent bowel surgery, and in any case the avoidance of taking SPS at the same time as any other oral medications [39]. These important limitations, together with the scarce data on SPS efficacy, may explain why there has been a need to develop new drugs for treating hyperkalemia by increasing GI potassium elimination.

## 6. Hyperkalemia: New Treatment Options

### 6.1. Patiromer

Patiromer FOS (for oral suspension), formerly known as RLY5016, was approved by the FDA in the USA in 2015, for the treatment of hyperkalemia. 

Patiromer is a cross-linked polymer of 2-fluoro acrylic acid (91%), with divinylbenzenes (8%) and 1,7-octadiene (1%). It is used in the form of its calcium salt (ratio 2:1) and with sorbitol (one molecule per two calcium ions or four fluoroacrylic acid units, corresponding to 4 g of sorbitol for each 8.4 g of patiromer); a combination called patiromer sorbitex calcium [49]. 

It appears as a dry powder for oral suspension, made of insoluble, spherical beads, with an average particle size of ≈ 100 µm. Pharmacokinetic analysis in animals showed that patiromer is not absorbed from the gut, is not metabolized, and is excreted in an unchanged form in the feces [50]. Patiromer works by binding the free potassium ions in the gastrointestinal tract, mainly in the distal colon lumen, and releasing calcium ions for exchange, thus lowering the amount of potassium available for absorption and increasing the amount that is excreted via the feces. The net effect is a reduction of the potassium levels in the blood serum. In CKD patients, it has been demonstrated that patiromer at a dose of 8.4 g twice a day lowered potassium levels within 7 hours of administration. These levels continue to decrease for at least 48 hours if treatment is continued, and remain stable for 24 hours after the administration of the last dose [51].

Because of its delayed onset of action (4–7 h), patiromer cannot be used as an emergency treatment for hyperkalemia [52].

### 6.2. Efficacy Data

Under in vitro conditions mimicking the pH and potassium content of the colon, patiromer binds 8.5–8.8 mmol of potassium per gram of polymer, which is a 1.5- to 2.5-fold improvement over the other polymers. In 33 healthy volunteers, 4.2, 8.4 and 16.8 g of patiromer, administered for 8 days three times a day, caused a dose-dependent increase in fecal potassium excretion (all *P* < 0.02 vs. placebo), with a corresponding dose-dependent reduction in urinary extraction [53].

A similar effect was also found in small cohorts of CKD and hemodialysis hyperkalemic patients, including those receiving RAASi [54]. However, the efficacy, safety and tolerability of patiromer were also tested in large clinical trials, which enrolled patients at high risk of hyperkalemia, such as patients with CHF, diabetes and CKD (see Table 1).

The first study exploring the efficacy and safety of patiromer in a large population was the "Evaluation of Patiromer in Heart Failure Patients" (PEARL HF) study, which was a 4-week, multicenter, double-blind, placebo-controlled study designed to evaluate the use of patiromer in the prevention of hyperkalemia. A total of 105 normokalemic patients (K^+^ 4.3–5.1 mmol/L) with CHF and either i) a history of hyperkalemia resulting in the discontinuation of ACEi/ARB/MRA and/or beta-blockers, or ii) CKD (eGFR < 60 mL/min) treated with one or more CHF therapies (ACEIs/ARBs and beta-blockers), were randomized to undertake double-blind treatment with 30 g/day patiromer or a placebo for 4 weeks, in association with spironolactone (at the initial dose of 25 mg/day, increased to 50 mg/day on day 15 if K^+^ was ≤ 5.1 mmol/L) [55].

The endpoints included the change in serum K^+^, the proportion of patients with hyperkaliemia (K^+^ > 5.5 mmol/L) and the proportion titrated to spironolactone 50 mg/day. At the end of treatment, compared with the placebo group, the group on patiromer showed significantly lowered serum K^+^ levels (−0.45 mmol/L, *P* < 0.001), a lower incidence of hyperkaliemia, and a higher proportion of patients on spironolactone 50 mg/day. Interestingly, in patients with CKD (*n* = 66), the difference in K^+^ levels between groups was −0.52 mmol/L (*P* = 0.031), and the incidence of hyperkaliemia was 6.7% for patiromer vs. 38.5% for the placebo. Adverse events were mainly gastrointestinal, and mild or moderate in severity.

Furthermore, the AMETHYST-DN study was a multicenter, open-label, dose-ranging, phase 2 trial that evaluated the efficacy of patiromer in the treatment of hyperkalemia in type 2 diabetic patients, with diabetic nephropathy and CKD and receiving RAAS inhibitors (ACEi and/or ARB for at least 28 days) [56]. The primary endpoint was potassium reduction, from baseline to week 4 or before the start of dose titration. The mean age was 66 years, 86% of patients had CKD stage 3–4, and 35% had CHF. Hyperkalemic patients at screening were immediately randomized into the treatment phase, while normokalemic patients were re-evaluated after the adjustment of antihypertensive therapy with the addition of losartan and/or spironolactone. Overall, 306 hyperkalemic patients (serum K^+^ 5–6 mmol/L) were eligible, and were stratified by potassium level into the categories of mild (5–5.5 mmol/L) and moderate (5.5–6 mmol/L) hyperkalemia, before being randomized to receive patiromer at increasing dosages (4.2 g, 8.4 g or 12.6 g bid in mild hyperkalemia, and 8.4 g, 12.6 g and 16.8 g bid for moderate hyperkalemia). The dosage was titrated to achieve a target serum K^+^ ≤ 5 mmol/L. 

Patiromer significantly reduced serum potassium levels from the baseline in all patients in a similar manner for the different doses, regardless of the initial potassium levels and independently of other comorbidities, such as CHF, advanced CKD or resistant hypertension. Moreover, in all patients the potassium lowering began ≈ 48 h after starting the patiromer, while target levels were reached early by patients with mild hyperkalemia. Interestingly, the reduction in serum K^+^ was achieved at week 8 (end of treatment phase) and maintained up to 52 weeks in patients who continued the treatment, whereas after discontinuation, serum potassium levels significantly increased. Regarding the safety profile, hypomagnesemia (7.2%) was the most common side effect, while constipation (6.3%) was the most common gastrointestinal adverse event. Moreover, patiromer treatment was also evaluated in a phase 3 study with CKD patients. So, in the OPAL-HK study, 237 patients with CKD stage 3–4 and serum potassium level of 5.1–6.5 mmol/L, undergoing stable treatment with one or more RAASi, were divided into two groups: those with mild hyperkalemia (serum K^+^ 5.1–5.5 mmol/L) that received patiromer 4.2 g bid, and those with moderate to severe hyperkalemia (5.5–6.5 mmol/L) that received 8.4 g bid [57]. Then the patiromer dosage was titrated to reach and maintain a potassium level of 3.8–5.1 mmol/L, and the patients were followed-up for 4 weeks. The authors found that patiromer significantly lowered potassium levels from baseline to week 4 in the whole study population, and for all prespecified subgroups (age < or > 65 years, presence/absence of diabetes or CHF, and maximal or submaximal dose of RAASi). Notably, at week 4, 76% of the overall population reached the target serum potassium level. After this first phase, the study proceeded with a randomized phase, in which patients with a baseline potassium level of 5.5–6.5 mmol/L and who achieved a target serum potassium level were randomized to continue the same dosage of patiromer or switch to placebo, and were followed-up for an additional 8 weeks. Furthermore, in this case, patiromer showed its efficacy, since, unlike the patiromer group, patients taking the placebo presented a significant increase in serum K^+^ (median K^+^ increase of +0.72 mmol/L). This difference was also observed across the prespecified subgroups of patients (regardless of age, gender, baseline K^+^ levels, diabetes, CHF and maximal/not maximal RAASi dosage) [58]. Moreover, a post-hoc analysis showed that the patiromer K-lowering efficacy and safety profile in CKD patients was not compromised by diuretic therapy [59].

An interesting opportunity offered by the potassium-lowering effects of patiromer has been explored in the recent phase 2 randomized AMBER study, which evaluated whether the use of patiromer allows a more persistent use of spironolactone in patients with CKD (eGFR 25 to ≤ 45 mL/min) and resistant hypertension [60]. 295 patients were randomly assigned to receive either placebo or patiromer (8.4 g once daily), in addition to open-label spironolactone (starting at 25 mg once daily). At week 12, 98 (66%) of the 148 patients in the placebo group, and 126 (86%) of the 147 patients in the patiromer group, remained on spironolactone, suggesting that patiromer can enable more patients to continue treatment with spironolactone under conditions in which this drug may be beneficial.

Although many data have been reported on the efficacy and safety of treatments with patiromer, on the other hand, several clinical studies are ongoing concerning the evaluation of patiromer in specific clinical settings. This is the case for the DIAMOND study, a phase 3b placebo-controlled and randomized trial, the intent of which is to determine if the patiromer treatment of CHF subjects with hyperkalemia while receiving RAASi allows the continued use of RAASi medications. Interestingly, this study will consider primary "hard" endpoints, constituted by the time to the first occurrence of cardiovascular death or hospitalization. The completion date of this trial is estimated as the middle of 2022 [61].

### 6.3. Safety and Tolerability

Patiromer was generally well tolerated. Overall, treatment-related adverse effects reported in the clinical trials occurred in ≈ 20% of the patients included.

They include electrolyte disorders, such as hypomagnesemia and hypokalemia, and mild gastrointestinal symptoms, such as constipation (8%), diarrhea (5%), nausea and flatulence [52]. In the product labeling, hypomagnesemia and hypokalemia are reported as adverse reactions in 5.3% and 4.7% of the treated patients, respectively [62].

Monitoring of serum magnesium is recommended, considering supplementations for patients who develop hypomagnesemia while on patiromer.

No cases of intestinal necrosis have been reported, probably as a consequence of the optimized characteristic of patiromer (i.e., uniform spherical shape, defined polymer bead size, low swelling ratio), which may improve the GI tolerability of this drug [63].

However, the use of patiromer is discouraged in patients with severe constipation, bowel obstruction or impaction, including abnormal post-operative bowel motility disorders, because of the potential ineffectiveness and the possibility of worsening gastrointestinal conditions [62].

### 6.4. Dosage, Administration and Drug Interactions

Based on the above-mentioned large trials, patiromer is recommended at a starting dosage of 8.4 g once daily, administered orally, which can be increased by 8.4-g increments per week, titrated up to a maximum of 25.2 g once daily. 

There are limited data on the use of patiromer for dialysis patients. As such, currently, no dose adjustment is advised. Patiromer presents as a powder that can be mixed with water, apple juice or cranberry juice. It should be mixed in an initial volume of 40 mL of water, then stirred and more water added to obtain the desired consistency. Then, the mixture should be taken within 1 hour of initial suspension, and its results are equally effective and well-tolerated when taken without food or with food [64].

Finally, it should be underlined that in vitro studies indicated the possibility that patiromer may interact with some medications. In particular, in studies of healthy volunteers, the use of patiromer decreased the systemic exposure of coadministered ciprofloxacin, levothyroxine and metformin [65]. For these reasons, the administration of other oral medications at least 3 hours before or 3 hours after patiromer is recommended.

## 7. Sodium Zirconium Cyclosilicate

Sodium zirconium cyclosilicate (SZC), formerly known as ZS-9, is an insoluble, inorganic, non-polymer zirconium silicate compound, comprising units of oxygen-linked zirconium and silicon atoms in the form of a microporous cubic lattice framework. 

It works as a selective cation exchange agent, primarily releasing hydrogen and sodium and preferentially capturing potassium, thus increasing its fecal excretion [66].

Its selectivity for potassium, which is > 25 times greater than that for calcium and magnesium ions, is due to the size of the pore, which is similar in diameter to unhydrated potassium (approximately 3 Å). Because of its high selectivity for potassium, SZC may bind it throughout the entire GI tract, and may exert a rapid K-lowering effect. It has been estimated that one gram of SZC binds about 3 mmol of potassium, and its activity begins within 1 h of the consumption [67].

At this stage, there are no studies comparing the pharmacodynamics properties of SZC when administered with or without food. Clinical studies have demonstrated that SCZ was not systemically absorbed, and no differences in urine and blood concentration were detected between treated and untreated patients. 

### 7.1. Efficacy Data 

SZC has been primarily evaluated in four randomized trials (ZS-002, ZS-003, ZS-004 and ZS-004E) and one open-label long-term study (ZS-005) (Table 2). 

The ZS-002 was a phase 2 study investigating the safety, tolerability, efficacy and pharmacodynamics of SZC in 90 patients with stage 3 CKD and hyperkalemia (K^+^ 5.0–6.0 mmol/L), who were randomized to receive SZC 0.3, 3 or 10 g three times a day, or a placebo. SZC showed a dose-dependent effect, and potassium levels significantly declined in the first 48 hours of the patients taking SZC at the doses of 3 g and 10 g (*P* = 0.048 and *P* < 0.0001, respectively, versus placebo) [68].

Then, SZC was also investigated in larger phase 3 randomized trials, where it showed a significant superiority to the placebo in achieving and maintaining normal serum potassium levels [69].

In particular, in the ZS-004 (Hyperkalemia Randomized Intervention Multidose ZS-9 Maintenance, HARMONIZE) trial, SZC safety and efficacy was tested in 258 patients with hyperkaliemia (K^+^ > 5.1 mmol/L), who initially received SZC 10 g three times a day for 48 h [70]. Then, those achieving normokalemia (*N* = 237) were randomized to receive SZC 5, 10 or 15 g once daily, or a placebo for the next 28 days (double-blind maintenance phase). Both initial and maintenance phases were characterized by a significant dose-dependent reduction of K^+^ in all SZC groups, compared with the placebo, even in the prespecified subgroups (CHF, diabetes, CKD and patients on RAASi). Compared with all other study groups, during the maintenance phase, there was a higher incidence of generalized and peripheral oedema in the SZC 15 g group (14.3%). 

Designed as an open-label extension of the HARMONIZE trial, the ZS-004E study investigated the safety and efficacy of SZC in patients with hyperkalemia who completed ZS-004, or who discontinued ZS-004 due to hypokalemia or hyperkaliemia in the maintenance phase and had a mean K^+^ of 3.5–6.2 mmol/L [71]. For 11 months, 123 patients received additional open-label treatment with SZC 10 g a day as an initial dose, which was then then titrated to maintain K^+^ 3.5–5.0 mmol/L. During the study period, a serum potassium value ≤ 5.1 mmol/L (primary endpoint) was achieved in 100% of THE patients, and K^+^ ≤ 5.5 mmol/L in 88.3%.

The long-term efficacy and safety of SZC were also investigated in the ZS-005 trial, a phase 3, prospective, open-label, single-arm,12-month study, in which 751 outpatients with hyperkalemia (K^+^ > 5.1 mmol/L) were enrolled [72]. No dietary restrictions or changes in RAASi therapy were required. The starting dosage was SZC 10 g thrice daily, for 24 to 72h (correction phase), then those who reached serum K^+^ 3.5–5.0 mmol/L at any point during the correction entered the maintenance phase (starting dose of SZC 5 g once daily). Dose titration (up to a maximum of 15 g daily, down to a minimum of 5 g every other day) was allowed based on serum potassium measurements. During the correction phase, 99% of patients achieved K^+^ 3.5–5.5 mmol/L, while the proportions of patients who achieved mean K^+^ ≤ 5.1mmol/L and ≤ 5.5 mmol/L across the maintenance were 88% and 99%, respectively.

Interestingly, a post hoc analysis of ZS-005 focused on the study of the subgroups of patients with CKD. Furthermore, in this case, SZC use was associated with a significant reduction in serum K^+^ levels in the long-term maintenance phase, in a similar manner even when patients were stratified via baseline-estimated glomerular filtration rate (i.e., eGFR < 30 or > 30 mL/min) [73].

The recent phase 3, randomized, double-blind HARMONIZE-Global trial examined the efficacy and safety of SZC among outpatients with hyperkalemia, from diverse geographic and ethnic origins [74].

A total of 248 patients achieving normokalaemia following a 48-h correction phase, with thrice-daily SZC 10 g, were randomized to once-daily SZC 5 g, SZC 10 g or placebo during a 28-day maintenance phase. Both initial and maintenance SZC regimens were associated with a significant reduction in K^+^ levels when compared to baseline values and placebo, and this effect lasted over the 28 days of treatment.

However, besides general studies on the treatment of chronic hyperkalemia, SZC has also been tested in acute and specific clinical settings. So, based on the pharmacokinetics data and the findings of clinical trials that reported a rapid effect of SZC in lowering serum K^+^, the authors of the ENERGIZE study explored the use of SZC in the ED [75]. It was a phase 2, multicenter, randomized, double-blind, placebo-controlled study, in which 70 patients with serum K^+^ > 5.8 mmol/L admitted at the ED were randomized 1:1 to SZC 10 g or placebo, administered up to three times during a 10-h period, in association with insulin and glucose. Reductions in K^+^ levels at 1 hour with SZC or the placebo were similar, probably due to the predominant potassium-lowering effect of the concomitant insulin and glucose treatment. A greater reduction in mean K^+^ from the baseline was observed in the SZC group, compared with the placebo at 2 hours (−0.72 vs. −0.36 mmol/L, respectively), suggesting that SZC may provide an incremental benefit in the emergency treatment of hyperkalemia.

However, the K^+^ level’s reduction was not significantly different between the SZC and placebo groups when they were evaluated 4 h after drug consumption.

Instead, the authors of the DIALIZE study tested the capacity of SZC to reduce blood potassium levels among patients undergoing HD [76].

So, they performed a phase 3b, double-blind, randomized trial, in which 197 patients on maintenance HD and predialysis hyperkalemia were randomized to receive a placebo or SZC 5 g once daily, on non-dialysis day, and were titrated to maintaining normokalemia over 4 weeks in increments of 5 g, up to a maximum of 15 g. The primary efficacy outcome involved the proportion of patients maintaining pre-dialysis serum K^+^ levels of 4–5 mmol/L, on three out of four dialysis treatments, after long interdialytic and not receiving rescue treatment. At the end of the study, 40 patients of the 97 receiving SZC (41.2%) met the primary endpoint, compared with 1 patient out of the 99 on placebo (1%). Interestingly, adverse effects, including interdialytic weight gain, were similar between the two groups. Thus, these findings suggest that SZC is an effective and well-tolerated treatment for predialysis hyperkalemia in HD patients (Table 2).

Further information on specific patient populations will be expected from the results of the ongoing PRIORITIZE-HF trial, which will evaluate SZC vs. placebo in patients with CHF taking RAASi. The completion date of this trial is estimated as the end of 2020 [77].

### 7.2. Safety and Tolerability

SZC is generally well tolerated. Hypokalemia occurred in 5.8% of patients enrolled in the ZS-005 trial [72].

In phase 2 and 3 trials, the incidence of gastrointestinal adverse events (nausea, constipation, vomiting or diarrhea) was similar between the treated group and the placebo group [78]. However, as was the case for patiromer, SZC should also not be used in patients with severe constipation, bowel obstructions or impaction, including abnormal postoperative bowel motility disorders [79].

A dose-related mild to moderate edema was observed in the SZC during the maintenance period (mostly in patients receiving maximum SZC dosage), but it was resolved spontaneously or with diuretic therapy. So, it is recommended to monitor signs of edema, especially in patients at risk of fluid overload, such CKD and CHF patients, probably adjusting dietary salt intake and the dose of diuretics [79]. 

Finally, a non-clinically relevant QTc interval prolongation, without an increased rate of arrhythmia, has been reported in some cases, probably as a consequence of the rapid decrease in serum potassium levels [69].

### 7.3. Dosage, Administration and Drug Interactions

The recommended starting dosage of SZC is 10 g three times a day; then, once normokalaemia is achieved (usually in 24–48 hours), the maintenance dosage is 5 g daily (the dosage can be titrated up to a maximum of 10 grams once daily, or down to a minimum of 5 g every other day). 

For patients on dialysis, SZC should only be given on non-dialysis days at starting doses of 5 g once daily, followed by titrating the dose according to the pre-dialysis serum potassium value after the long inter dialytic interval [79].

SZC presents as a powder, and before consumption, the entire content of a sachet should be mixed with approximately 45 mL of water, and stirred well. It can be taken with or without food [66]. If hyperkaliemia persists after 72 hours with the maximum dosage, other treatment approaches should be considered.

SZC can transiently increase gastric pH, potentially affecting the absorption of co-administered drugs that exhibit pH-dependent solubility.

In vivo studies in healthy volunteers showed that, when co-administered with SZC, there was an increase in systemic exposure to weak acids, such as furosemide and atorvastatin, and a decrease in systemic exposures to weak bases, such as dabigatran [80].

So, the general advice is that other oral medications should be administered at least 2 hours before or 2 hours after SZC.

## 8. Conclusions

For decades, the absence of a therapeutic alternative to SPS has represented one of the main limitations to the management of hyperkalemia, especially in patients at high risk, such as those with CHF, diabetes and CKD undergoing treatment with RAASi. 

Therefore, the development of new potassium-lowering agents, such as patiromer and SZC, has offered new opportunities for improving the management of hyperkalemia, even considering that, unlike SPS, these medications have proven their efficacy in large clinical trials in different clinical settings (see Table 3). Remarkably, patiromer and SZC appear to be well tolerated and safer compared to SPS, with the report of only mild GI disorders and no cases of intestinal necrosis.

However, although the available data are encouraging and support the use of patiromer and SZC in the management of hyperkalemia, several important issues remain to be explored [81].

For example, there are no data on compliance with the treatment, and no study has yet directly compared the efficacy and tolerability of patiromer with SZC. 

Moreover, one of the main barriers to the use of the new potassium-lowering agents may be constituted by the higher cost of these treatments compared to SPS. There is thus a need to perform accurate cost-effectiveness analyses, also to evaluate the economic effects of the implementation of these new treatments. These analyses should consider the potential benefits derived from the reduced incidence of adverse effects, and from the optimization of chronic RAASi treatment, which, in turn, may improve clinical outcomes for CHF and CKD patients.

In this regard, it has been demonstrated by mathematical models that hyperkalemia prevention and treatment with patiromer is a potentially cost-effective intervention for the long-term maintenance of RAASi in patients at risk of hyperkalemia [82].

So, several studies are ongoing, and others should be designed to define the potentiality offered by the application of these new potassium binders in specific clinical settings, and to elucidate their roles in improving long-term clinical outcomes.

## Figures and Tables

**Table 1:** Main clinical trials evaluating use of patiromer for chronic hyperkalemia.

Study, Year	Study Population	*N*	Study Design (with Patiromer Dosage)	Follow-Up (Weeks)	Main Results
**PEARL-HF** **2012 [55]**	CHF, CKD or previous hyperkalemia causing RAASi interruption plus indication to start spironolactone	105	Randomized and double blind: patiromer 15 g bid vs. placebo Spironolactone starting dose 25 mg, progressive dose titration	4	Mean K^+^ reduction: −0.45 mmol/L patiromer vs. placebo (*P* < 0.001)
**AMETHYST-DN** **2015 [56]**	Diabetes plus CKD (stage 3–4) receiving RAASi with known hyperkalemia or those who developed hyperkalemia during run-in phase	306	Randomized and open label. Patients on ACEi or ARB started on spironolactone (1) Mild HK (5.1–5.5 mmol/L): Patiromer 4.2–8.4–12.6 g bid (2) Moderate HK (5.6–5.9 mmol/L): Patiromer 8.4 g– 12.6 g–16.8 g bid	52	(1) Mild HK: K^+^ reduction − 0.35 mmol/L for 4.2 g, −0.51 mmol/L for 8.4 g, −0.55 mmol/L for 12.6 g (2) Moderate HK: K^+^ reduction −0.87 mmol/L for 8.4 g −0.97 mmol/L for 12.6 g −0.92 for 16.8 g
**OPAL-HK** **2015 [57]**	CKD patients (stage 3–4) on RAASi	243	Initial treatment phase: (1) Mild HK (K^+^ 5.1–5.5 mmol/L) Patiromer 4.2 g bid (2) Moderate HK (K^+^ 5.6 –5.9 mmol/L) Patiromer 8.4 g bid	4	Mean K^+^ reduction: −1.01 mmol/L vs. basal values
		107	Randomized maintenance phase: Continue patiromer (*n* = 55) vs. placebo (*n* = 52)	8	K^+^ increase: +0.72 mmol/L in placebo vs. 0 mmol/L in patiromer (*P* < 0.001)

Abbreviations: CHF, Chronic heart failure; CKD, Chronic Kidney Disease; HK, Hyperkalemia; RAASi, Renin-Angiotensin-Aldosterone system inhibitors; bid, twice a day.

**Table 2:** Main clinical trials evaluating the use of sodium zirconium cyclosilicate for acute and chronic hyperkalemia.

Study, Year	Study Population	*N*	Study Design (with SCZ Dosage)	Follow-Up (Weeks)	Main Results
**HARMONIZE** **2014 [70]**	K^+^ > 5.1 mmol/L 69% CKD	258	Open-label: SZC 10 g tid No control group	48 h	Normokalemia (K^+^ 3.5−5 mmol/L): 84% at 24 h 98% at 48 h
		237	Randomized normokalemic pts: Placebo vs. SZC 5 g–10 g–15 g single dose	4	Normokalemia: Placebo 46% SZC 5 g: 80%, SZC 10 g: 90% SZC 15 g: 94% (*p* < 0.001 vs. placebo for all SZC)
**ZS-005** **2019 [72]**	K^+^ ≥ 5.1 mmol/L 74% CKD	751	Open-label: SZC 10 g tid	72 h	Normokalemia (K^+^ 3.5–5 mmol/L): 99%
		746	Maintenance phase SZC 5 g daily, titrated to 5g–15g No control group	52	K^+^ < 5.1 mmol/L: Overall 88% months 3–12 (466 pts completed the trial)
**ENERGIZE** **2020 [75]**	K^+^ ≥ 5.8 mmol/L in ED	7	Randomized: SZC 10 g (*n* = 38) vs. Placebo (*n* = 32) up to 3 times in 10 h + (glucose-insulin)	1	Mean change in K^+^ at 4 h: −0.41 mmol/L SZC −0.27 mmol/L placebo Mean change in K^+^ at 2 h: −0.72 mmol/L SZC −0.36 mmol/L placebo
**DIALIZE** **2020 [75]**	HD patients with predialysis K^+^ ≥ 5.4 mmol/L		Randomized: SZC 5–15 g single dose in non-dialysis days vs. Placebo	8	K^+^ 4–5 mmol/L: SZC group 41.2% placebo group 1.0%

Abbreviations: SZC, Sodium Zirconium Cyclosilicate; CHF, Chronic heart failure; CKD, Chronic Kidney Disease; HK, Hyperkalemia; RAASi, Renin-Angiotensin-Aldosterone system inhibitors; ED, Emergency Department; HD, hemodialysis; tid, three times a day.

**Table 3:** Main characteristics of the approved potassium binders for the treatment of hyperkalemia.

Drug, FDA Approval	Mechanisms Location	Onset of Action	Patient Groups Tested in Clinical Trials	Adverse Effects	Cost
SPS, 1958	Non-specific organic ion-exchange resin. It exchanges sodium for Potassium. Colon	Variable, hours to days [39]	CKD, HD	Mild to moderate gastrointestinal effects, including colonic necrosis, poor tolerability, electrolyte disorders	Low
Patiromer, 2015	Non-specific organic ion-exchange resin. It exchanges calcium for potassium. Colon	Within 7 h [51]	CHF, Diabetes, CKD +/- mRAASi	Mild gastrointestinal effects, hypomagnesaemia, hypokalemia (3–6%)	Very high
SZC, 2018	Selective inorganic non-polymer. It exchanges sodium and hydrogen for potassium. Entire gastrointestinal tract	Median time 2 h [69]	CHF, CKD, HD ED +/- RAASi	Mild gastrointestinal effects, oedema and hypokalemia (dose-dependent)	Very high

Abbreviations: SPS, Sodium Polystyrene Sulfonate; SZC, Sodium Zirconium Cyclosilicate; CHF, Chronic heart failure; CKD, Chronic Kidney Disease; HK, Hyperkalemia; RAASi, Renin-Angiotensin-Aldosterone system inhibitors; ED, Emergency Department; HD, hemodialysis.
